# Dysfunctional LAT2 Amino Acid Transporter Is Associated With Cataract in Mouse and Humans

**DOI:** 10.3389/fphys.2019.00688

**Published:** 2019-06-04

**Authors:** Emilia Boiadjieva Knöpfel, Clara Vilches, Simone M. R. Camargo, Ekaitz Errasti-Murugarren, Andrina Stäubli, Clara Mayayo, Francis L. Munier, Nataliya Miroshnikova, Nadège Poncet, Alexandra Junza, Shomi S. Bhattacharya, Esther Prat, Vanita Berry, Wolfgang Berger, Elise Heon, Anthony T. Moore, Óscar Yanes, Virginia Nunes, Manuel Palacín, Francois Verrey, Barbara Kloeckener-Gruissem

**Affiliations:** ^1^Institute of Physiology, University of Zurich, Zurich, Switzerland; ^2^Zurich Center for Integrative Human Physiology, University of Zurich, Zurich, Switzerland; ^3^Swiss National Centre of Competence in Research Kidney.CH, University of Zurich, Zurich, Switzerland; ^4^Genes, Disease and Therapy Program, Molecular Genetics Laboratory – IDIBELL, Barcelona, Spain; ^5^U730 and U731, Centro de Investigación Biomédica en Red de Enfermedades Raras, Barcelona, Spain; ^6^Institute for Research in Biomedicine, The Barcelona Institute of Science and Technology, Barcelona, Spain; ^7^Institute of Medical Molecular Genetics, University of Zurich, Zurich, Switzerland; ^8^Department of Biology, ETH Zurich, Zurich, Switzerland; ^9^Jules-Gonin Eye Hospital, University of Lausanne, Lausanne, Switzerland; ^10^Metabolomics Platform, IISPV, Department of Electronic Engineering, Universitat Rovira i Virgili, Tarragona, Spain; ^11^CIBER of Diabetes and Associated Metabolic Diseases (CIBERDEM), Madrid, Spain; ^12^Andalusian Molecular Biology and Regenerative Medicine Centre – CABIMER, Seville, Spain; ^13^UCL Institute of Ophthalmology, London, United Kingdom; ^14^Genetics Section, Department of Physiological Sciences, Faculty of Medicine and Health Sciences, University of Barcelona, Barcelona, Spain; ^15^Neuroscience Center Zurich – ZNZ, University of Zurich and ETH Zurich, Zurich, Switzerland; ^16^Department of Ophthalmology and Vision Sciences, The Hospital for Sick Children, Toronto, ON, Canada; ^17^Moorfields Eye Hospital, London, United Kingdom; ^18^Department of Ophthalmology, School of Medicine, University of California, San Francisco, San Francisco, CA, United States; ^19^Departament de Bioquímica i Biomedicina Molecular, Facultat de Biologia, Universitat de Barcelona, Barcelona, Spain

**Keywords:** amino acid transporters LAT2 and TAT1, gene expression, cataract, ocular tissues, mouse model, patient screen

## Abstract

Cataract, the loss of ocular lens transparency, accounts for ∼50% of worldwide blindness and has been associated with water and solute transport dysfunction across lens cellular barriers. We show that neutral amino acid antiporter LAT2 *(Slc7a8*) and uniporter TAT1 (*Slc16a10*) are expressed on mouse ciliary epithelium and LAT2 also in lens epithelium. Correspondingly, deletion of LAT2 induced a dramatic decrease in lens essential amino acid levels that was modulated by TAT1 defect. Interestingly, the absence of LAT2 led to increased incidence of cataract in mice, in particular in older females, and a synergistic effect was observed with simultaneous lack of TAT1. Screening *SLC7A8* in patients diagnosed with congenital or age-related cataract yielded one homozygous single nucleotide deletion segregating in a family with congenital cataract. Expressed in HeLa cells, this LAT2 mutation did not support amino acid uptake. Heterozygous LAT2 variants were also found in patients with cataract some of which showed a reduced transport function when expressed in HeLa cells. Whether heterozygous LAT2 variants may contribute to the pathology of cataract needs to be further investigated. Overall, our results suggest that defects of amino acid transporter LAT2 are implicated in cataract formation, a situation that may be aggravated by TAT1 defects.

## Introduction

Cataract, the condition of partial or complete loss of transparency in the ocular lens, is the most frequent cause for vision impairment worldwide (51%)^1^. Typically the condition affects individuals over 50 years of age (McCarty and Taylor, 2001) and since life expectancy is increasing the number of affected individuals is raising, for instance in the United States from 24.4 million in 2010 it will change to potentially 50 million in 2050^2^, making age-related cataract an important socio-economical problem. Age-related cataract is considered a multi-factorial disease where both genetic and environmental risk factors contribute to the pathogenesis (Shiels et al., 2010; Shiels and Hejtmancik, 2013, 2015). In contrast, early onset cataract, which is either present at birth or developing within the first few years of life, affects a minority of patients (∼3–6/10,000 births) (Graw, 2004; Shiels and Hejtmancik, 2013; Medsinge and Nischal, 2015). Usually this latter type follows Mendelian inherited patterns where X-linked recessive, autosomal dominant and autosomal recessive modes of inheritance have been observed in about 30 different causative genes (Shiels and Hejtmancik, 2013). Routinely, the affected lens is replaced by insertion of an artificial lens, but existing side effects (Grzybowski and Kanclerz, 2019; Zhang et al., 2019) motivate for the search of alternative, non-invasive therapeutic approaches. This requires detailed understanding of the pathophysiology.

**FIGURE 1:**
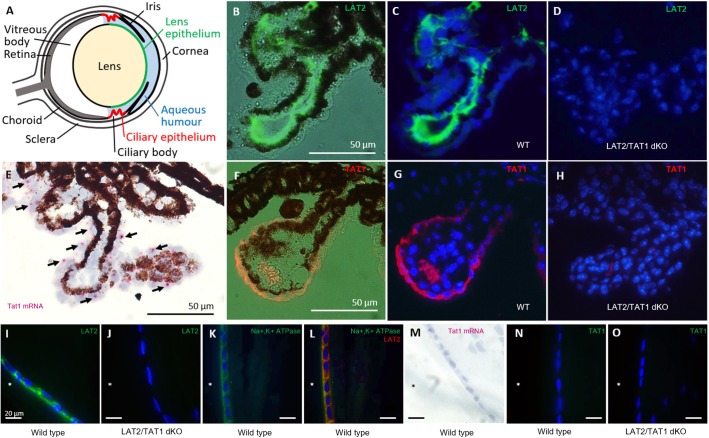
Localization of SLC7A8 (LAT2) and SLC16A10 (TAT1) amino acid transporters in ciliary and lens epithelia of the mouse eye. **(A)** Schematic drawing of the mouse eye. **(B–D)** Localization of LAT2 protein in ciliary epithelium. Paraffin sections of wild type (WT) mouse eyes were stained for LAT2 (green). **(B)** Fluorescence image is superposed to the bright field image and **(C)** nuclei are labeled with DAPI on a dark field. The green staining of LAT2 localizes to the basolateral side of the dark brown/black pigmented ciliary epithelial cells seen in the bright field **(B)**. **(D)** Absence of green LAT2 staining in the ciliary epithelium of a LAT2/TAT1 double KO mouse. **(E–H)** Localization of TAT1 in ciliary epithelium. **(E)** RNAscope^®^ signal of *SLC16A10* (*TAT1*) mRNA (red dots, black arrows) overlaying the non-pigmented ciliary epithelial cells on a bright field image taken from a wild-type mouse eye cryosection. Pigmented cells appear brown and black. **(F)** Fluorescence images of TAT1 (orange/red) superposed to bright field image and **(G)** together with nuclear DAPI staining on dark field. The orange/red TAT1 signal localizes to the basolateral side of the non-pigmented epithelial cells. The red/orange signal within the villus is due to erythrocytes. Panel **(H)** shows the absence of red TAT1 staining in the ciliary epithelium of a LAT2/TAT1 double KO mouse. **(I–L)** Localization of LAT2 in lens epithelium. **(I)** Cryosections of a wild type and **(J)** of a LAT2/TAT1 double KO mouse lens, incubated with anti LAT2 antibody (green) that labels the basolateral side of lens epithelial cells facing the anterior chamber (^∗^) of a wild type, but not of a LAT2/TAT1 double KO mouse. **(K)** Paraffin-embedded lens section of a wild type mouse on which Na,K-ATPase alpha subunit (green) and **(L)** Na,K-ATPase and LAT2 (red) are labeled. LAT2 is shown to co-localize with Na,K-ATPase (co-staining yellow) at the basolateral side of the lens epithelial layer. **(M–O)** Absence of TAT1 in lens epithelium. Panel **(M)** shows the absence of TAT1 mRNA signal by RNAscope^®^ (no pink dots), in a wild type mouse lens epithelium. **(N)** Absence of TAT1 immunofluorescence signal (green) on wild type and **(O)** LAT2/TAT1 double KO mouse lens cryosections.

**FIGURE 2:**
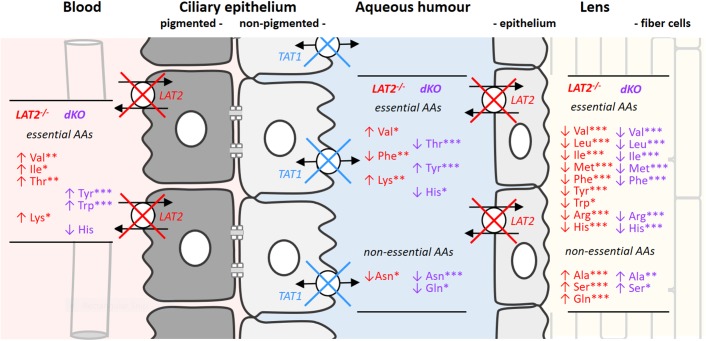
Changes in amino acid concentrations in serum, aqueous humor and lenses caused by the KO of LAT2 and the dKO of LAT2 and TAT1. Schematic drawing of ciliary and lens epithelia: The ciliary epithelium is composed of two cell layers facing each other with their apical sides that are linked by gap junctions. The cell layer at the vascular side forms the pigmented epithelium (gray) and the cell layer facing the aqueous humor the non-pigmented one (white) which, with its tight junctions prevents paracellular diffusion between the extracellular space of the ciliary body and the aqueous humor (see also Figure 1). LAT2 localizes to the basolateral surface of ciliary pigmented epithelial cells and to the basolateral side of lens epithelial cells, whereas TAT1 localizes to the basolateral side of the non-pigmented cell layer of the ciliary body and is absent from the lens. The impact of the single LAT2 (red) knockout and of the double KO of LAT2 (red) and TAT1 (blue) on amino acid concentrations in the different compartments is indicated as lists of amino acids that are significantly up- (↑) or down- (↓) regulated. Statistical significance of the difference with wild type calculated by one way ANOVA followed by Bonferroni post-test: ^∗^*P* < 0.05, ^∗∗^*P* < 0.01, ^∗∗∗^*P* < 0.001. The actual mean concentrations for all proteinogenic amino acids in the four genotypes is given in Supplementary Table S3.

The transparency of the lens is maintained by its unique physiology and anatomy of tightly packed highly elongated fiber cells, which lose their organelles during a differentiation process (Augusteyn, 2010) that continues throughout the entire lifetime resulting overall in minimal cellular turnover and likely in the accumulation of metabolic defects that could lead to loss of transparency. As the lens lacks vasculature, alternative routes for the supply of nutrients and metabolites from the bloodstream to the interior of lens cells are required. In support, the lens is in immediate contact with the aqueous and vitreous humors. For molecular transport, the ciliary epithelium represents a first barrier localized between the extracellular space of the ciliary body and the aqueous humor. This epithelium is composed of two epithelial cell layers, the pigmented and the non-pigmented ones, facing each other with their apical membranes and connected with gap junctions (Raviola and Raviola, 1978; Figure 1A, 2). Thus, transepithelial transport of water, ions and nutrients relies on transporters expressed in the plasma membranes localized on both sides of the paracellular barrier formed by tight junctions that link non-pigmented epithelial cell layer together.

A second cellular barrier between blood and lens interior is formed by the lens epithelium that localizes between the aqueous humor and the extracellular fluid of the lens and the tightly packed fiber cells. Water and solutes have thus to cross the lens epithelial cells and the plasma membrane of lens fiber cells. These latter cells express next to AQP0 water channel (aquaporin) (Zampighi et al., 2002) and connexin (Cx) hemichannels (Shi et al., 2018) also some solute transport proteins (Truscott, 2000). Accordingly in the lens, that includes also the aqueous humor-facing lens epithelium, a variety of transport proteins have been localized, for instance in addition to aquaporins (Barandika et al., 2016) also ion channels (K^+^, Na^+^, Ca^2+^), pumps (Na,K-ATPase) (Mathias and Rae, 2004), glucose transporters (GLUT1, GLUT3, and SGLT2) (Merriman-Smith et al., 1999, 2003), creatine transporter CRT2 (MCT12) (Abplanalp et al., 2013) and amino acid transporters (GLYT AND ASCT2) (Marcantonio and Duncan, 1983, 1987; Lim et al., 2006, 2007). These latter transporters need to import all amino acids involved in lens cell metabolism and in particular also those required for the synthesis of the tripeptide glutathione (GSH), which is a vital antioxidant known to be important for the preservation of lens transparency (Giblin, 2000). Another amino acid that plays a particular role in the lens is L-tryptophan which is the precursor of several UV filter compounds such as 3-hydroxykynurenine and 3-hydroxyanthranilic acid (Wood and Truscott, 1993). The transport of aromatic amino acids like L-tryptophan and of other large neutral amino acids across the ciliary epithelium and into the lens presumably involves at least two transporters, namely LAT2 (Q9UH15, encoded by *SLC7A8*, Gene ID: 23428; OMIM 604235) and TAT1 (MCT10, encoded by *SLC16A10*). LAT2 is a L-type amino acid transporter associated with the glycoprotein CD98hc (4F2hc, encoded by *SLC3A2*), which exchanges all neutral amino acids against each other with high affinity at the cell exterior and independent of sodium (Pineda et al., 1999; Rossier et al., 1999; Meier et al., 2002). In contrast, the T-type amino acid transporter TAT1, which is also expressed in the eye^3^, is a uniporter that has a narrow selectivity range for aromatic amino acids (Kim et al., 2001; Ramadan et al., 2006). Based on their abundant co-localization at the basolateral membrane of the proximal kidney tubule and the small intestine and also on functional experiments performed in *Xenopus laevis* oocytes, LAT2 and TAT1 have been suggested to cooperate functionally for neutral amino acid (re)absorption (Pineda et al., 1999; Rossier et al., 1999; Makrides et al., 2014). In a recent study, we could verify and further extend this hypothesis using a double KO (dKO) mouse model (Vilches et al., 2018). Importantly, these transporters are also expressed in sensory organs and defects of LAT2 have been shown to be involved in age-related hearing loss (Espino Guarch et al., 2018). The high level of *SLC7A8* expression in the eye and in particular in the lens (see text footnote 3), supported our hypothesis that mutations affect lens transparency, potentially also with the cooperation of uniporter TAT1. Here we report association of cataract with LAT2, based on a homozygous knockout mouse model (*Slc7a8*^−/−^) (Lat2 KO) (Font-Llitjós, 2005) in which lens amino acid concentrations are affected. In accord, we identified a homozygous mutation in *Slc7A8* segregating in a family with congenital cataract. Whether the heterozygous sequence alterations that were found in patients with congenital and age-related cataract represent risk factors will be discussed. Furthermore, in the mouse model that simultaneous lacks LAT2 and TAT1, the occurrence of cataract is increased, suggesting synergistic effects. Taken together, a model emerges proposing that defects of LAT2-mediated amino acid transport can influence cataract formation.

## Materials and Methods

### *In situ* RNA Hybridization

RNA molecules were detected *in situ* by using the RNAscope^®^ technology (Advanced Cell Diagnostics, Newark, CA, United States) according to manufacturer’s guidelines. Whole eyes were fixed in 3% PFA for 24 h and paraffin embedded. 5 μm sections were mounted on Superfrost Plus slides and deparaffinized before being pre-treated as follows: 10 min in Hydrogen Peroxide, 5 min incubation in the boiling Target Retrieval Solution and 30 min incubation with Protease Plus at 40°C. The slides were then subjected to the RNAscope^®^ 2.5 HD Assay-Red, strictly following manufacturer’s instructions, using the probe named Mm-Slc16a10-O1 targeting 483–1560 of NM_028247.4. At the end of the procedure, slides were counterstained, dried in a 60°C oven for 1 h, dipped in Xylene and mounted with VectaMount (Vector Laboratories, Burlingame, CA, United States). Sections were imaged using a brigthfield microscope (Nikon Eclipse TE300, Nikon Instruments Inc., Melville, NY, United States).

### Immunofluorescence

Eyes were extracted as described below and fixed in 3% paraformaldehyde (PFA) for 24 h embedded in paraffin blocks. Lenses were snap frozen and later mounted in OCT. Paraffin sections of the whole eye (5 μm) and cryosections of the lenses (6 μm) were prepared. Sections were incubated with primary antibodies (diluted in PBS, 2% BSA, 0.04% Triton X-100) overnight at 4°C. Subsequently, sections were incubated with the appropriate secondary antibodies (Supplementary Table S1) and mounted in Glycergel (DakoCytomation, Denmark). Sections were viewed on a Nikon Eclipse TE300 epifluorescence microscope (Nikon Instruments Inc., Melville, NY) equipped with a DS-5M Standard charge-coupled device camera (Nikon Instruments Inc.) and acquired with NIS Elements (Nikon Instruments Inc.). Images were merged using Photoshop 9.

### Collection of Mouse Serum, Aqueous Humor and Lenses for Amino Acid Quantification by Ultra-Performance Liquid Chromatography (UPLC)

Wild type, heterozygous and homozygous mice were anesthetized with Attane^TM^ (Isoflurane, Piramal Critical Care, Germany) for heart blood collection. Immediately after this terminal bleeding, the aqueous humor was collected using a 30-gauge needle and the lenses were isolated as described below. Amino acid analysis was performed at the Functional Genomics Center Zurich (FGCZ). Samples were deproteinized with sulfosalicylic acid (5% final concentration), and amino acid concentrations were determined using the MassTrak Amino Acid Analysis Solution method (Waters, Milford, United States) according to the manufacturer’s instructions.

### Mouse Models and Cataract Scoring

Genetically modified animals involved in this study included LAT2 KO mice (Font-Llitjós, 2005) and TAT1 KO (Mariotta et al., 2012); both are loss-of-function models [*Slc7a8* (LAT2) with deletion of exon 1 and *Slc16a10* (TAT1) with non-sense mutation leading to a premature stop at position 88]. Additionally, LAT2/TAT1 double knockout (dKO) mice were generated by crossing TAT1 KO with LAT2 KO animals. Both knockout models have pure C57BL/6 genetic background. It has been reported that the C57BL/6J genetic background carries the potential for embryonic developmental eye problems, which may result in anophthalmia, microphthalmia and corneal and lenticular opacities at low frequency (Pierro and Spiggle, 1967; Kalter, 1968; Cook and Sulik, 1986). Therefore, the statistical evaluation was in comparison to wild type (WT) siblings. Animals were anesthetized with Attane^TM^ and immediately euthanized by terminal bleeding via cardiac puncture. Eyes were then extracted and kept in ice-cold PBS solution on ice during the entire procedure. Lens of animals aged between 2 and 19 months were removed either through the opened cornea or posteriorly through the opened eye near the optic nerve. As we did observe some developmental morphological abnormalities of the lens as well as normally shaped lenses with opacities, the number of animals that was investigated in our study was increased. In the control group of 97 WT eyes, cataract was observed in 3%. Cataract (opacities in the lens) was scored first *in vivo*, then *ex vivo* after eye dissection, followed by a microscopic analysis (Leica Z16 APO) of the lens. Lenses were placed on EM copper grid, 200/inch mesh, in order to evaluate their transparency. To avoid biased analysis, cataract assessment was performed blinded with respect to the animal genotype, age and gender. Animals were bred at two facilities: at the University of Zurich, Switzerland and at the IDIBELL in Barcelona, Spain. Food and water were given *ad libitum*.

### Patients Diagnosed With Cataracts

Patients’ cataract condition and their DNA samples were provided from three clinical sources: Switzerland (family D and 132 patients with juvenile cataract and 360 patients with age-related cataracts, all diagnosed by Dr. F. Munier), the United Kingdom (95 samples from probands, all with autosomal dominant inherited childhood cataracts, diagnosed by Dr. A. T. Moore), and Canada (81 patients with cataracts of various age of onset, diagnosed by Dr. E. Heon).

### DNA Sequence Analysis of SLC7A8 Exons

Genomic DNA from patients diagnosed with either juvenile or age-related cataract was amplified using primers for coding exons 1 to 11 of the gene *SLC7A8* (Entrez ID 23428, NM_22244.3, OMIM 604235) (Sequence and annealing temperature in Supplementary Table S2). PCR amplification of 35 cycles, 1 min for each step was followed by Sanger sequencing reactions using ABI chemistry (BigDye version 1), hardware (DNA analyzer 3730 or Genetic Analyzer 3130xl and software (SeqScape) (Applied Biosystems, Risch-Rotkreuz, Switzerland). PCR and sequencing of samples showing rare sequence variants was repeated. Variants were annotated using Alamut-HT (Interactive Biosoftware, Rouen, France). Frequencies were taken from dbSNP and Exome Aggregation Consortium (ExAC) data base (May 2018) (for web site links see below).

### Site-Directed Mutagenesis

The QuikChange site-directed mutagenesis kit (Agilent, Santa Clara, CA, United States) was used to introduce point mutations in LAT2 sequence, according to the manufacturer’s protocol. The pcDNA3.1-StrepTag fused LAT2 construct (N-terminally tagged) was used as template (Costa et al., 2013). Amino acid substitutions were introduced into LAT2 sequence using a compatible reverse primer and forward primers (Supplementary Table S2). All primers annealed to the coding sequence, and the position of the mutated codon was underlined. All constructs were verified by DNA sequencing and then used for transient transfection.

### Cell Culture and Transfection

HeLa cells were maintained at 37°C/5% CO_2_ in Dulbecco’s modified Eagle’s medium (Life Technologies, Carlsbad, CA, United States) supplemented with 10% (v/v) fetal bovine serum, 50 units/ml penicillin, 50 μg/ml streptomycin, and 2 mM L-glutamine. HeLa cells were transiently co-transfected with plasmid constructions mentioned (0.2 μg cDNA/well of 24-well plate) above (or empty vector for mock cells) and human His-CD98hc (His-4F2hc) (N-terminally tagged) cloned in pcDNA4His-MaxC (0.2 μg cDNA/well of 24-well plate) (Fort et al., 2007) with the use of Lipofectamine 2000 (Invitrogen, Carlsbad, CA, United States) following the manufacturer’s protocol as described (Rosell et al., 2014). Amino acid transport and fluorescence microscopy analyses were carried out 48 h after transfection.

### Amino Acid Transport Assay

Amino acid uptake was measured by exposing replicate cultures at room temperature to [^3^H]-labeled alanine, [^3^H]-tryptophan or [^3^H]-isoleucine (1 μCi/ml; Perkin Elmer, Waltham. MA, United States) in sodium-free transport buffer (137 mM choline chloride, 5 mM KCl, 2 mM CaCl_2_, 1 mM MgSO_4_, and 10 mM HEPES, pH 7.4). Initial rates of transport were determined using an incubation period of 1 min and 50 μM of cold amino acid. Assays were terminated by washing with an excess volume of chilled transport buffer. Cells were lysed using 0.1% SDS and 100 mM NaOH. Then 200 μL were used for radioactivity counting in a Packard Tri-Carb Liquid Scintillation Counter, and 15-μL duplicates were used to determine protein content using the Pierce BCA kit, Pierce. Uptake values were corrected by cellular protein in all cases, and by the expression of Strep Tagged LAT2 by fluorescence microscopy (Total corrected Cellular Fluorescence, TCCF; see next section) for all constructs except for mutant F436Sfs^∗^22 that showed compromised expression compared with reference LAT2.

### Visualization of Strep-Tagged Amino Acid Transporters by Fluorescence Microscopy

To analyze the effect of the mutations on LAT2 protein expression and plasma membrane localization, fluorescence microscopy of Strep-tagged reference and mutant transporters was performed on a semi confluent monolayer of transfected HeLa cells cultured on glass coverslips. Glass coverslip-grown cells were incubated with 1 mg/ml wheat germ agglutinin (WGA) labeled with Texas-Red (Thermo Fisher Scientific, Waltham, MA, United States) at 37°C for 10 min, rinsed three times with phosphate-buffered saline-Ca^2+^-Mg^2+^ and fixed for 15 min in 4% paraformaldehyde. Fixed cells were blocked in blocking buffer (10% FBS and 0.1% saponin in PBS) for 1 h and then incubated for 1 h with primary antibody (anti-Strep Tag GT517, 1/100; Abcam, Cambridge, United Kingdom). Secondary goat-anti-mouse-FITC antibody (Life Technologies, Carlsbad, CA, United States) was incubated for 2 h protected from light and rinsed three times with phosphate-buffered saline. Nuclear staining was performed by incubating 1 μg/ml Hoechst (Thermo Fisher Scientific, Waltham, MA, United States) for 10 min, rinsed three times with phosphate-buffered saline and then mounted with aqua-poly/mount coverslipping medium (Polysciences, Inc., Warrington, PA, United States). Images were taken using a Nikon E1000 upright epifluorescence microscope. All images were captured during 200 ms with the exception of those corresponding to p.Phe436Serfs^∗^22 that was overexposed to 2 s, to reveal the subcellular localization of this very low expressing variant. To quantify LAT2 reference control and mutated transporters expression levels in cells, a single in-focus plane was acquired. Using ImageJ (v1.48, NIH), an outline was drawn around each cell and area and mean fluorescence measured, along with several adjacent background readings. The total corrected cellular fluorescence (TCCF) = integrated density – (area of selected cell × mean fluorescence of background readings), was calculated (Conway et al., 2016).

### Statistics

Pooled data are shown as means ± SEM (n) where n represents the number of independent experiments (*n* = 3 for uptake experiments in HeLa cells, passage 10–12 in the laboratory) in each of 4 replicas. For statistical comparison, ANOVA (Analysis of Variance) followed by Bonferroni post-test or Two-tailed *t*-test were performed using a statistical software package (Prism v. 5.0–7.0, GraphPad, San Diego, CA, United States) or R, a free software for statistical computing. Odds ratio and Fisher Exact Probability Test were calculated using the online calculator from Vassar College^4^.

### Study Approval

All animals were kept in accordance with the Swiss and Spanish federal law, respectively, and experiments were performed with the approval of the Swiss Veterinary Council and with the approval of the Animal Experimentation Ethics Committee at the IDIBELL (AAALAC accredited facility, B99000010) and by the corresponding department of Generalitat of Catalunya (DAAM#3866). Patients’ participation in this study was approved by the respective ethics regulatory bodies and all patients gave written informed consent. Experiments respected the principles expressed in the Declaration of Helsinki.

### Web Resources

Web sites for sequence information and prediction programs.

Alamut Visual vs2.7.2, a mutation analysis software^5^. DNA and protein sequence information GeneCards http://www.genecards.org/ and NCBI http://www.ncbi.nlm.nih.gov/ and UniProtKB http://www.uniprot.org/uniprot. SNP variant information: ESP Exome variant server http://evs.gs.washington.edu/. ExAC http://exac.broadinstitute.org/ and Ensemble v83 http://www.ensembl.org/index.html. Gene expression https://genome.uiowa.edu/otdb/, https://genevestigator.com/gv/ and https://mae.hms.harvard.edu/. Online calculator from Vassar College (see text footnote 4).

## Results

### Expression and Localization of Slc7a8 (LAT2) and Slc16a10 (TAT1) Amino Acid Transporters

To determine the localization of the neutral amino acid exchanger LAT2 and the aromatic amino acid uniporter TAT1 in mouse eyes, we performed immunofluorescence and RNA *in situ* hybridization experiments on tissue sections of the ciliary body and the ocular lens from wild type and knockout (KO) animals (Figure 1). On the ciliary body of wild type animals, LAT2 protein was detected at the basolateral side of the pigmented epithelial layer, which forms the blood side border of the double-layered ciliary epithelium (Figure 1B,C). The specificity of this strong signal was confirmed by its absence in LAT2/TAT1 double KO animals (Figure 1D). In contrast, TAT1 was detected at the mRNA and protein levels in the non-pigmented cell layer of the ciliary epithelium that faces the aqueous humor (Figure 1E–G). The absence of TAT1 protein in the ciliary bodies of LAT2/TAT1 double KO confirmed antibody specificity (Figure 1H). Interestingly TAT1 protein appeared to localize to the aqueous humor facing basolateral side of these cells. Within the lens, a strong LAT2 signal localized to the basolateral side of the lens epithelium where it co-localized with the Na,K-ATPase (Figure 1I,K,L). Also in this case the specificity of the signal was confirmed by its absence in lenses of double KO animals (Figure 1J). In contrast, TAT1 was absent from the lens both at the mRNA level (no hybridization signal in Figure 1M and no qPCR signal, data not shown) and at the protein level (no immunofluorescence signal, Figure 1N,O). These LAT2 and TAT1 expression patterns observed in mouse eye fully correspond to the mRNA expression levels reported for human eye, namely equally high levels of LAT2 and TAT1 mRNA in ciliary body and a very high level for LAT2 and a near-absence of TAT1 in the lens^6^. No significant difference of the staining pattern was observed in heterozygous and wild type animals (Supplementary Figure S1). In summary, the expression of LAT2 or TAT1 on both sides of the ciliary epithelium and of LAT2 on the aqueous humor-facing basolateral side of the lens epithelium support the hypothesis that these transporters play an important role for the transport of amino acids from the blood to the lens and thus for lens amino acid homeostasis.

### Effect of LAT2 and TAT1 Knockout Mutations on Amino Acid Composition in Serum, Aqueous Humor and Lens in Mice

Disruption of the neutral amino acids exchange performed by the antiporter LAT2 and of the facilitated diffusion of aromatic amino acids mediated by the uniporter TAT1 has been shown to affect not only the homeostasis of immediate transporter substrate amino acids but also to strongly impact the general amino acid balance (Vilches et al., 2018). Such profound misbalance of amino acid concentrations is what we observed when we analyzed amino acid levels in the serum, aqueous humor and lens from LAT2 and double KO mice (Figure 2 and Supplementary Table S3) and compared them with findings from wild type animals. Figure 2 displays the sequential barriers that limit the circulation between blood and lens, namely the double layered ciliary epithelium that separates the blood side extracellular space from the aqueous humor and the lens epithelium, which forms the barrier toward the lens extracellular space. As previously described, the lack of LAT2 slightly influences the concentration of some serum amino acids (Val, Ile, Thr, and Lys levels significantly increased), whereas the additional absence of TAT1 leads to a strong and significant increase of the aromatic amino acids Tyr and Trp, as in TAT1 single KOs (Mariotta et al., 2012; Vilches et al., 2018). More striking, amino acid concentration changes relative to the wild type situation are observed in the aqueous humor, which is largely produced by the ciliary epithelium that lacks LAT2 on the blood facing side (Figure 2) and, in the case of the double KO, also TAT1 on the aqueous humor facing basolateral side. Interestingly, although the amino acid concentration changes were significant in both genotypes, they were quite different from each other. In contrast, the impact of the lack of LAT2 was clearly dominant in the lens, as amino acid levels were quite similarly affected in double KOs. Importantly, the concentration level of nearly all essential amino acids was strongly decreased in lenses of both genotypes (statistically significant decrease in LAT2 KO of Val, Leu, Ile, Met, Phe Tyr Trp, Arg, and His) whereas some non-essential amino acids were increased (Ala, Ser, and Gln). Interestingly, the decrease of essential amino acids was quantitatively even more pronounced in double KO lenses where in most cases levels were reduced to <50% of the control, whereas amino acid levels were not altered in LAT2 heterozygous mice (Supplementary Table S4). Taken together, these results show that the absence of LAT2 leads to a strong imbalance of the normal amount of amino acids in the lens, which is further exacerbated when TAT1 is missing. They further suggest, that only severely reduced levels of functional transporter may lead to a phenotype and that a single functional LAT2 allele under certain physiological conditions may support normal lens physiology.

### Cataracts in Slc7a8 (LAT2), Slc16a10 (TAT1) and Double KO Mice

**FIGURE 3:**
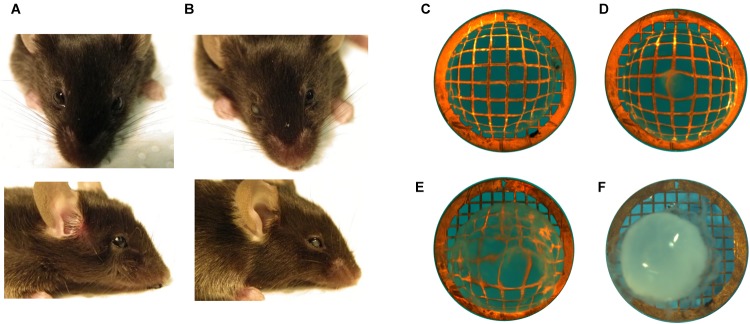
Assessment of cataract in mice. Examples of mice **(A)** without cataract showing top and side view and **(B)** with unilateral (right eye) cataract. Microscopic view of extracted lenses placed on copper grid (200/inch mesh) to visualize opacities with examples of **(C)** a transparent lens, **(D)** mild opacity, **(E)** intermediate opacity, and **(F)** mature cataract.

**FIGURE 4:**
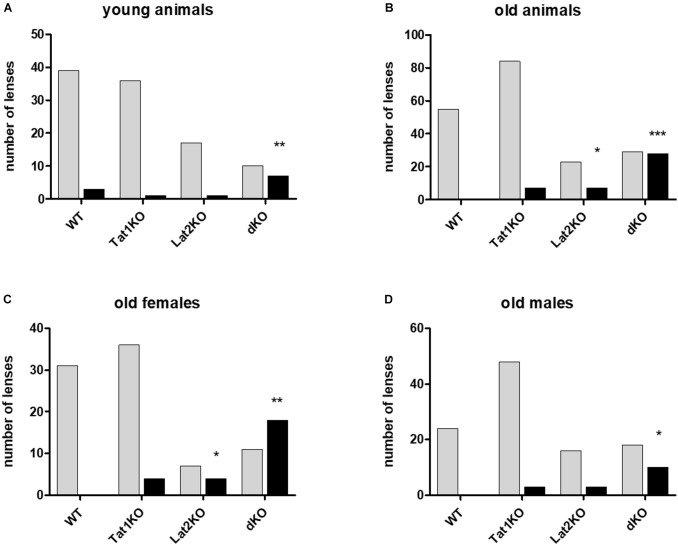
Effect of genotype on cataract formation in mice. Genotypes are given on X-axis: wild type (WT), single (TAT1 and LAT2 knockouts) and double KO. Number of lenses assessed on Y-axis with either cataracts (black bars) or no cataract (light bars). **(A)** Young animals; **(B)** Old animals; **(C)** Old females; **(D)** Old males. For statistical evaluation mutants were compared to wild type (WT) animals. Statistical significance is displayed as: ^∗^*P* < 0.05, ^∗∗^*P* < 0.01, ^∗∗∗^*P* < 0.001, ^∗∗∗∗^*P* < 0.0001 (see Supplementary Table S5).

A possible impact of LAT2 and TAT1 knockout on the prevalence of cataract phenotype was evaluated. Initial assessment of lens opacities was performed in the living animal (Figure 3A,B). Upon sacrificing the animals, eyes were enucleated and the lenses were inspected microscopically for transparency (Figure 3C) and varying degrees of opacities (Figure 3D–F). Mature cataract was often associated with reduced size of the lens (Figure 3F). The result of this morphological assessment of lenses taken from knockout mice lacking LAT2 (LAT2 KO) either alone or in combination with the TAT1 (dKO) was compared to that of their wild type (WT) littermates and of TAT1 KO mice. A total of 97 WT, 48 LAT2 KO, 128 TAT1 KO, and 74 dKO eyes were examined (Supplementary Figure S2). The number of cataract occurrence per genotype is shown in Figure 4 and statistical significance is based on odds ratio calculations. In comparison to the wild type animals, the absence of LAT2 (LAT2 KO) alone and together with that of TAT1 (dKO) resulted in a statistically significant higher frequency of cataract occurrence in old animals (*p* = 0.016 and *p* = 0.0012, respectively), specifically in old females (*p* = 0.0187 and *p* = 0.0017, respectively) (Figure 4 and Supplementary Table S5), suggesting that the loss of LAT2 represents a risk factor for cataract in aging animals. Increased frequency of cataract was observed when TAT1 was also missing (dKO) and affected both age groups and sexes. However, the lack of TAT1 KO alone did not seem to influence cataract formation. Taken together, simultaneous absence of both transporters leads to increased occurrence in cataract in all groups, while the lack of LAT2 alone was sufficient to result in a significant increase of cataracts in old animals, in particularly in old females.

### Screening of Sequence Variants in the Coding Region of SLC7A8 in Patients With Cataracts

**FIGURE 5:**
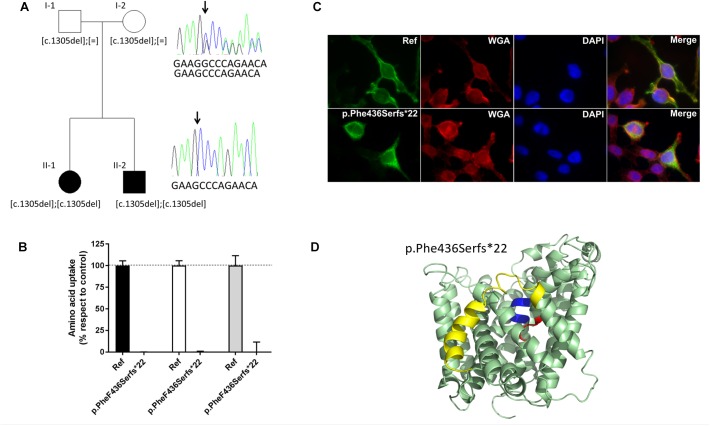
*SLC7A8* mutation Phe436Serfs^∗^22 in cataract patients and its effect on function. **(A)** Pedigree (two generation family) of proband with congenital cataract. Both affected children (II-1 and II-2) (filled symbol) carry a homozygous deletion of nucleotide c.1305C leading to the Frameshift p.Phe436Sers^∗^22. Both unaffected parents (unfilled symbol) are heterozygous for the mutation. Arrow points to the position of the deletion. The reference sequence in the electropherogram shown reads 5′-GAAGGCCCAGAACA-3′. **(B)** Alanine (black bars), tryptophan (white bars), and isoleucine (gray bars) uptake of cataract associated sequence variant F436Sfs^∗^22 in SLC7A8, tested in HeLa cells. Uptake mean and standard error of mean (SEM) were calculated in relation to the reference uptake. **(C)** Subcellular localization of Strep tagged hLAT2 and cataract variant pPhe436Serfs^∗^22 in HeLa cells. Green staining: LAT2, red staining: wheat germ agglutinin, blue (DAPI) staining: nuclei and merged showing co-localization with the plasma membrane marker for reference LAT2 but not for pPhe436Serfs^∗^22, which does not co-localize with plasma membrane, indicating that this variant is retained intracellularly. **(D)** Location of the frameshift mutation Phe436Serfs^∗^22 within the structure of human LAT2. Cartoon representation of the structural homology model of human LAT2 (Rosell et al., 2014) in an outward-facing conformation, based on the atomic structure of the bacterial homolog AdiC (Gao et al., 2010; Kowalczyk et al., 2011). Unwound segments of TM domains 1 (TM1) and TM6 that interact with the α-amino-carboxyl end of the amino acid substrates are colored in blue and red, respectively. The frameshift mutation Phe436Serfs^∗^22 produces a new 21 amino acid long carboxy terminus that replaces the yellow labeled last 100 amino acids that encompass for instance the end of TM11 and the last TM domain of LAT2 (TM12).

Based on the strong association of *Slc7a8* with cataract in the mouse model, *SLC7A8* became a potential candidate gene for human cataract as well. Furthermore, the statistically non-significant different results of amino acid concentrations in wild type and heterozygous LAT2 mutants (Supplementary Table S4) suggested to search in particular for homozygous sequence alterations. Although the observations in mice suggested a stronger association with late- than early onset cataract, we included 308 patients with childhood (congenital) cataract and 360 patients diagnosed with age-related cataract (ARC) in the screen for sequence variants in the coding region and canonical splice sites of *SLC7A8*. We found one homozygous alteration: a deletion of one nucleotide leading to a Frameshift and a premature termination toward the carboxy terminus of the translated protein (c.1305del; p.Phe436Serfs^∗^22). In the SNP databases, this variant (rs778197019) was not found in the coding transcript of SLC7A8; only in a non-coding transcript variant, without indication of its frequency (Supplementary Table S6). In the family both affected siblings (II-1 and II2 in Figure 5A) are homozygous for this variant while the parents are heterozygous. Consanguinity was not indicated.

The proband (II-1 in Figure 5A) had been diagnosed with bilateral congenital sutural and zonular cataract, which was operated at 6 and 8 weeks of age. While a younger sibling was also diagnosed with this cataract, both parents were unaffected.

### Functional Studies for Uptake of Amino Acids

This homozygous alteration was reconstructed and tested in the human HeLa cell model for amino acid uptake. Both, the reference (wild type) and the variant LAT2 catalytic light chains were co-expressed with the glycoprotein subunit [CD98hc (4F2hc)] to increase their potential plasma membrane localization (Rosell et al., 2014). The lack of membrane staining upon expression of the variant LAT2 is in line with the observed lack of amino acid transport (Figure 5B,C).

Taken together, the functional tests of the LAT2 mutation in Hela cells combined with the cataract diagnosis of the two homozygous patients strongly suggest a correlation in humans between LAT2 defect and cataract.

## Discussion

We have shown that the lack of functional LAT2 provokes a strong decrease of lens essential amino acid levels in mice and furthermore associates with cataracts in both, mice and humans. The localization of LAT2 on the blood facing side of the pigmented ciliary epithelium and on the aqueous humor-facing side of the lens epithelium explains the strong decrease in lens essential amino acid content observed in LAT2 KO mice (Figure 1, 2). Also, the fact that LAT2 functions as amino acid antiporter mediating the influx of extracellular essential amino acids mainly in exchange with the efflux of highly concentrated intracellular non-essential amino acids (Pineda et al., 1999; Meier et al., 2002) agrees with the observed increase of some non-essential amino acids in the lens of LAT2 KO mice. The localization of TAT1 at the aqueous humor side of the ciliary epithelium is also in line with the effects observed at the level of the lens essential amino acids in the double KO mice, where we observed mostly a stronger decrease than in the LAT2 KOs (Supplementary Table S3). It has to be mentioned though that TAT1 KO *per se* leads to elevated plasma aromatic amino acids, explaining the less drastic effect of the double KO on this class of essential amino acids compared for instance to the branched chain ones.

As described above, the knockout of mouse LAT2 was associated with ARC, in particular in female animals, and the additional defect of TAT1 strengthened this association. Based on these results, it appears that their might be a causative link between the decrease in lens amino acids and the development of cataract. Indeed, an important function of the amino acids within the lens may be to serve the metabolic needs of the epithelium and the differentiated fiber cells (Swarup et al., 2018). These authors found for instance a substantial change in amino acid concentrations upon deletion of the energy providing glucose transporter GLUT1. The depletion of tryptophan, a specific essential aromatic amino acid, might play an important role for the pathogenesis of cataract. We report here a significant effect on tryptophan in the mouse eye, and in rats, it was shown that a tryptophan-restricted diet resulted in altered lens structure (McAvoy et al., 1979). Specifically, fiber cell differentiation was accompanied with slowing the process of nuclei breakdown (Vrensen et al., 2004). Furthermore, tryptophan was shown to play an important role in the lens’ response to UV light (Wood and Truscott, 1993) and it has long been known that exposure to UV light is an environmental risk factor for cataract (Roberts, 2001, 2011). Lens contain several molecules that absorb UVA light (320–400 nm) one of which is tryptophan-derived 3-hydroxykynurenine O-β-D-glucoside (3OHKG) (Gad et al., 2014). This protective molecule has been reported to decrease with age, potentially further promoting age-related loss of lens transparency.

Screening the LAT2 gene of 308 human patients with childhood (congenital) cataract and 360 with ARC we identified in two siblings with congenital cataract a homozygous LAT2 variant. This variant is a single nucleotide deletion leading to a Frameshift with premature termination of the translated protein (c.1305del; p.Phe436Serfs^∗^22) and its expression in HeLa cells showed a lack of surface expression and amino acid transport (Figure 5). In view of possible structural changes introduced by the frame shift, should the aberrant mRNA not be removed by non-sense mediated decay, LAT2 protein may present with an altered C-terminus starting at the extracellular end of *trans*-membrane domain TM11 and lacking TM12. It has to be mentioned that both heterozygous parents had not developed cataract at the time of the investigation. Furthermore, we identified six heterozygous variants in 10 other patients with congenital or childhood cataract and four variants in nine patients with ARC (Supplementary Figure S3 and Supplementary Table S6). Two of these variants (p.Ala94Thr and p.Ser29Phe) were found multiple times in both groups of cataract patients (Figure 5A and Supplementary Table S6). The frequency of these variants in each group is statistically not significantly different from each other (9/360 with ARC and 11/308 with early onset cataract; *p* = 0.2795).

Whereas the association of the homozygous mutation blocking LAT2 function with congenital cataract in two siblings strongly suggests a causative relationship between the LAT2 defect and cataract, it is less clear whether heterozygous variants of LAT2 identified in early and late onset cataract patients participate in the pathogenesis. Given our findings that in mice the heterozygous lack of LAT2 had no measurable effect on LAT2 expression in ciliary and lens epithelia observed by immunofluorescence nor on amino acid concentrations in aqueous humor and lens, it seems likely that only strong defects in amino acid transport may be a risk for cataract formation. Interestingly, testing the transport function of the heterozygous variants in HeLa cells showed that all but two of them transported the tested amino acids (Ala, Trp, Ile) either as well as wild type LAT2 or with a reduction of less than 50% (Supplementary Figure S3). Two variants, p.Val302Ile and p.Ala94Thr displayed a stronger reduction of transport specifically for the large aromatic amino acid Trp (∼75% reduction) and both map to the protein core of LAT2. Of note is that variant p.Val302Ile plays a role in age-related hearing loss in which it was shown to display a reduced transport rate of another aromatic amino acid, tyrosine (Espino Guarch et al., 2018). The fact that the variants reported here show no or little effect on the uptake of alanine and isoleucine in our HeLa cell model is interesting and worth further studies. Taken together, these functional experiments do, for most of the identified heterozygous variants, not provide strong support for their involvement in cataract pathogenesis. In contrast, for the two variants that display strongly decreased Trp transport implication in pathogenesis of cataract appears more plausible since low levels of tryptophan or of other essential amino acids may lead to accelerated lens aging and, as a possible consequence, to the development of cataract.

Nevertheless, as age related cataract is a multifactorial disease, interactions with other genes or factor are likely involved in the manifestation of the phenotype and in combination, single allele defects may contribute. The presence and hence possible effects of additional sequence variants in either known or yet unknown cataract genes in any of the patients cannot be excluded. A candidate gene for such additional variant is *SLC16A10* encoding the aromatic amino acid transporter TAT1, in particular since our mouse studies suggest synergistic effects of TAT1 and LAT2. In an initial search, we screened the DNA from patients with variants in *SLC7A8* for variants in *SLC16A10* and identified several, which require further functional analyzes in order to understand their possible molecular interactions (unpublished data).

The question of gender bias in cataract is difficult to assess and is only indirectly measured by the number of surgeries. For age-related cataract a preponderance of females has been reported (Rao et al., 2011), but reasons are likely complex and could be based among others on socio-economic and cultural conditions. Nevertheless, a genetic component may also influence the impact on gender and a possible factor could be related to the amino acid transporters LAT2 and TAT1 where we found a higher frequency of cataract among old females in the animal model, and moreover, all ARC patients with heterozygous *SLC7A8* variants were also females. Whether these genes carry genetic risk factors affecting a gender bias will await further studies. With respect to aging, the evidence provided here in combination with a recent report that LAT2 is involved in age-related hearing loss (Espino Guarch et al.), suggests that this gene likely influences a genetic risk for sensory processes during aging.

## Conclusion

In conclusion, the identification of sequence variants in the amino acid transporter LAT2 in mouse models and in humans supports *SLC7A8* as a novel cataract gene. The amino acid transporter TAT1 has a modifying influence on cataract formation as shown in double KO mice. Thus, it appears that amino acid imbalance could contribute to the explanation of a pathomechanism of lens opacification. It is expected that additional transporters with function in the lens will also be implicated in murine and human cataract.

## Data Availability

The raw data supporting the conclusions of this manuscript will be made available by the authors, without undue reservation, to any qualified researcher.

## Ethics Statement

Animals were bred at two facilities: at the University of Zurich, Switzerland and at the IDIBELL in Barcelona, Spain. At both locations, standar ethics procedures were observed. Patients’ written consent form has been collected.

## Author Contributions

BK-G, FV, MP, and VN: conceptual design and funding acquisition. EK, CV, SC, EE-M, AS, BK-G, FV, MP, VN, and SB: experimental design, data generation, interpretation. EK, CV, SC, CM, EE-M, NP, NM, EP, AS, AJ, BK-G, FV, MP, and VN: methodology. VN, MP, FV, and WB: recourses. SC, ÓY, BK-G, FV, MP, and VN: supervision. FM, VB, EH, and AM: clinical work. EK, EE-M, AS, NP, VN, MP, FV, BK-G, and SC: manuscript writing. All authors: revision and approval.

## Conflict of Interest Statement

The authors declare that the research was conducted in the absence of any commercial or financial relationships that could be construed as a potential conflict of interest.

## Footnotes

1 https://www.who.int/news-room/fact-sheets/detail/blindness-and-visual-impairment

2 http://www.nei.nih.gov/health

3 https://genome.uiowa.edu/otdb/

4 http://vassarstats.net/tu.html

5 http://www.interactive-biosoftware.com/alamut-visual/

6 http://genome.uiowa.edu/otdb
