# Supplementary material for: Dysfunctional LAT2 Amino Acid Transporter Is Associated With Cataract in Mouse and Humans
Source: Front Physiol. 2019 Jun 4;10:688. doi: 10.3389/fphys.2019.00688 (PMC6558864; doi:10.3389/fphys.2019.00688)
Supplement: Supplementary file 1 [file Presentation_1.pdf]

Supplemental Information: Figures

Figure S1. Expression and localization of amino acid transporter LAT2 in eye of wild type and heterozygous LAT2 KO mice. Lens (top row) and ciliary body (bottom row) of the mouse eye from wild type (left column), homozygous LAT2 KO (middle column) and heterozygous LAT2 KO (right column). Paraffin sections were stained for LAT2 (green) and nuclei were labeled with DAPI (blue) on a dark field.

Figure S2. Description of the mice population. Three knockout mouse models, all in C57BL/6J genetic background, TAT1 KO, LAT2 KO, and double KO (dKO), were compared to wild type (WT) littermates. (A) Distribution of the tested lenses by age groups (young animals are < 6 months of age; old animals are ≥ 6 months of age); (B) distribution of the tested lenses by sex.

Figure S3. Analysis of heterozygous SLC7A8 variants. (A) Alanine (black bars), tryptophan (white bars) and isoleucine (grey bars) uptake by variants of LAT2, tested in HeLa cells. Uptake mean and standard error of mean (SEM) were calculated in relation to the reference uptake. (B) Subcellular localization of Strep tagged hLAT2 variants in HeLa cells. Variants pArg8Pro, pGly18Arg, pSer29Phe, pAla94Thr, pMet291Ile, pVal302Ile and pSer487Asn are expressed at the plasma membrane. Green staining: LAT2, red staining: wheat germ agglutinin, blue (DAPI) staining: Merge displays co-localization of LAT2 with the plasma membrane marker for all constructions.

Figure S1
Localization of amino acid transporter LAT2 in mouse eye.

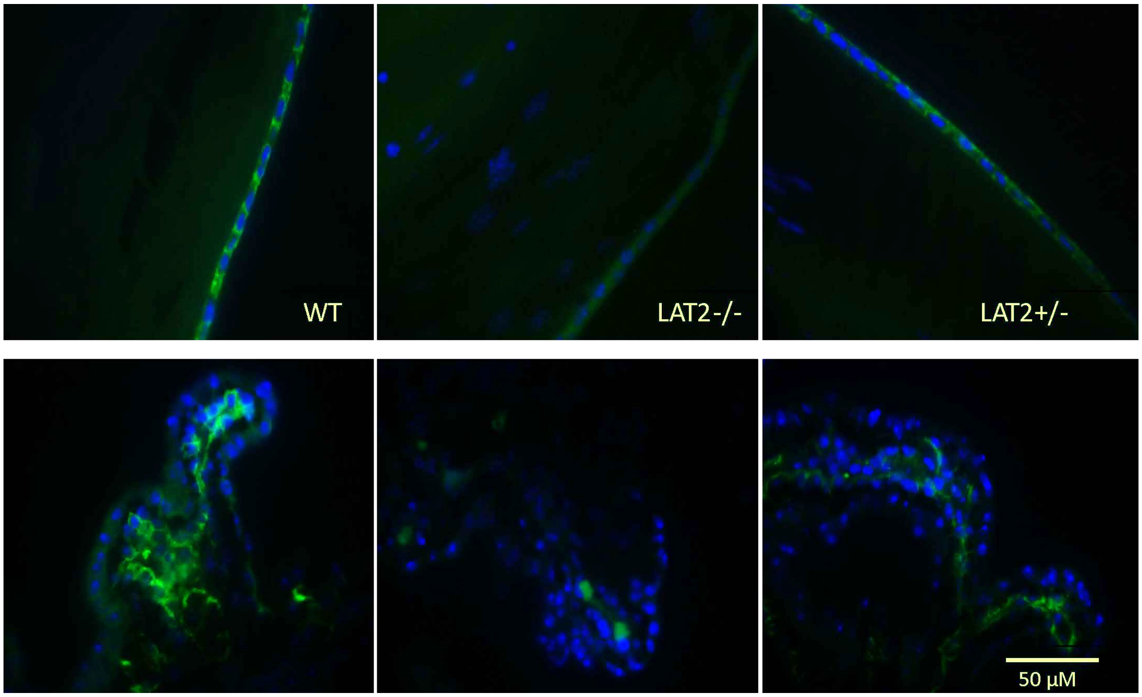

Figure S2
Description of the mouse population.

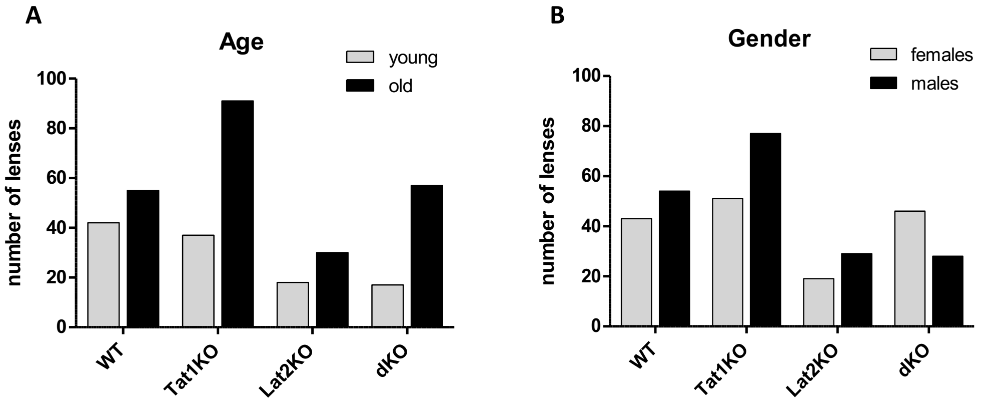

Figure S3
Effect of heterozygous sequence variants in SLC7A8 tested in HeLa cells

A

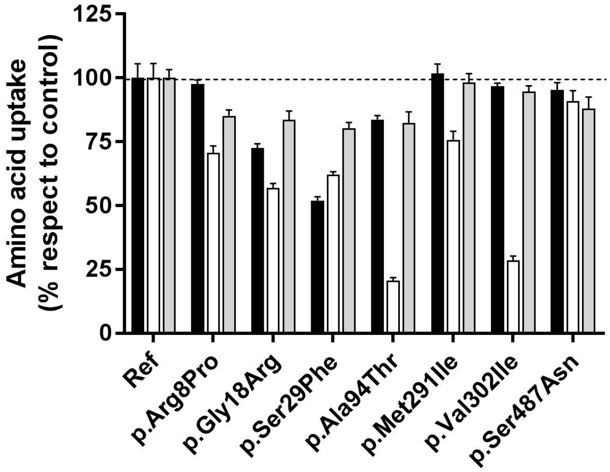

B

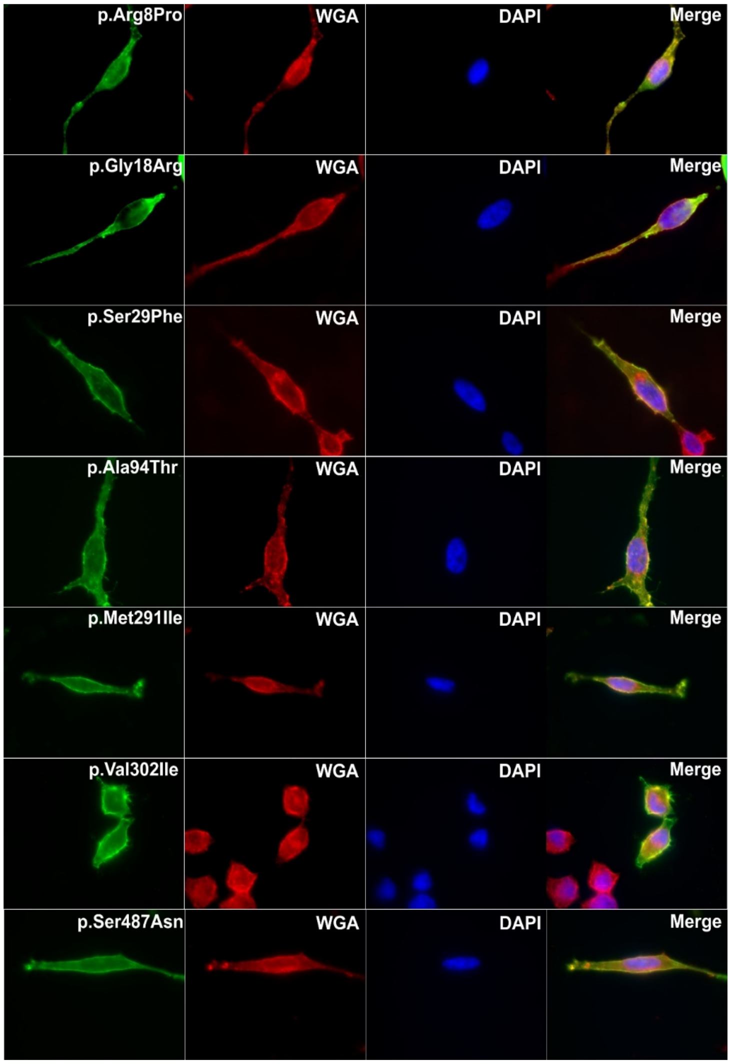

Supplemental Information: Tables

Table S1. Antibodies dilution scheme. Amino acid single letter nomenclature is given.

Table S2. Primer information. Sequence is given 5' to 3'. For site directed mutagenesis the position of the mutated codon is underlined.

Table S3. Amino acid concentration in serum, aqueous humor (AH) and lens from wild type (WT), TAT1 knockout (TAT1), LAT2 KO (LAT2) and TAT1/LAT2 double KO (dKO) mice. For statistical analysis, amino acid concentrations measured in different experimental series were each normalized to the mean of their wild type (wt) values. Genotypes were compared by one-way ANOVA, followed by Bonferroni posttest performed on all pairs of columns. Letters indicate which pair is statistically different: A=*LAT2*$^{-/-}$ versus wt, B=double KO versus wt, C=*LAT2*$^{-/-}$ versus *TAT1*$^{-/-}$, D=double KO versus *TAT1*$^{-/-}$, E=double KO versus *LAT2*$^{-/-}$, F=*TAT1*$^{-/-}$ versus wt, and the level of significance is indicated as *$P<0.05$, **$P<0.01$, ***$P<0.001$

Table S4. Amino acid concentration in serum, aqueous humor (AH) and lens from wild type (WT), LAT2 double knockout (LAT2 KO) and heterozygous LAT2 (LAT2-hetero) mice. For statistical analysis, amino acid concentrations measured in different experimental series were each normalized to the mean of their wild type (wt) values. Genotypes were compared by one-way ANOVA, followed by Bonferroni post-test performed on all pairs of columns. Letters indicate which pair is statistically different: A=*LAT2*$^{-/-}$ versus wt, B= *LAT2*$^{-/+}$ versus wt, C= *LAT2*$^{-/+}$ versus *LAT2*$^{-/-}$. The level of significance is indicated as *$P<0.05$, **$P<0.01$, ***$P<0.001$

Table S5. Statistical assessment of cataract development in mice population. Shown are frequency, odds ratio (OR), confidence intervals (CI), and statistical significance (P).

Table S6. Patients with *SLC7A8* sequence variants. Patient ID is assigned arbitrarily. rs numbers were listed whenever SNP information was available, otherwise "novel" was assigned. Minor allele frequency was taken from ExAC database for European including Finnish population and for total (all populations) (http://exac.broadinstitute.org/), SNP data base (https://www.ncbi.nlm.nih.gov/projects/SNP/) and exome sequencing project (http://evs.gs.washington.edu/EVS/). All variants were heterozygous, except **, which was reported from a non-coding transcript variant without frequency data (https://www.ncbi.nlm.nih.gov/snp/?term=rs778197019).

Table S1
Antibodies dilution scheme

Antigen	Primary antibody/ cell marker	Dilution	Antigen retrieval	Secondary antibody (Molecular Probes)
LAT2	CPIFKPTPVKDPDSE EQP Eurogentec (Seraing, Belgium)	1:1000	Cryosection, 0.1% SDS in PBS (5 min/RT) Paraffin, 10 mM Na-citrate pH6 (10min/98°C)	Alexa Fluor 488 donkey-anti-rabbit IgG (1:500) Alexa Fluor 594 donkey-anti-rabbit IgG (1:500)
TAT1	CGSSGIFKKDASII Genosphere (Paris, France)	1:500	Paraffin, 0.1% SDS in PBS (5 min/RT)	Alexa Fluor 488 goat-anti-rabbit IgG (1:500)
NaKATP ase	Santa Cruz, sc-48345	1:1000	Paraffin, 10 mM Na-citrate pH6 (10min/98°C)	Alexa Fluor 488 donkey-anti- mouse IgG (1:500)
DNA	DAPI, Merk	1:5000		-

Table S2
DNA Primer information

gene/exon/mutation	name	sequence	purpose
SLC7A8 (MIM604235) exon 1	SLC7A8_ex1.2__For	cgtgttctcttttatctgcttgc	screening
	SLC7A8_ex1_rev	aaacttgaccagtggaggtga	screening
SLC7A8 (MIM604235) exon 2	SLC7A8_ex2_for	gaaatgagaccaccctccaa	screening
	SLC7A8_ex2_rev	caaacctggtcctccatcat	screening
SLC7A8 (MIM604235) exon 3	SLC7A8_ex3.2_for	ttgtcactgcagagggctgg	screening
	SLC7A8_ex3.2_rev	gagctatgatcatgcggctgc	screening
SLC7A8 (MIM604235) exon 4	SLC7A8_ex4_for	ttgagcttgctggatgtctg	screening
	SLC7A8_ex4_rev	ccttcccttttgtcaagcaa	screening
SLC7A8 (MIM604235) exon 5	SLC7A8_ex5_for	tgttgggttcactggagaca	screening
	SLC7A8_ex5_rev	acggcaaatggtttaagtgc	screening
SLC7A8 (MIM604235) exon 6	SLC7A8_ex6_for	acacgcatgcagaaagacag	screening
	SLC7A8_ex6_rev	agaacttgggggtgatttcc	screening
SLC7A8 (MIM604235) exon 7	SLC7A8_ex7_for	catgcattaaccaccctgtg	screening
	SLC7A8_ex7_rev	cagcacacactcctcagcat	screening
SLC7A8 (MIM604235) exon 8	SLC7A8_ex8_for	gactggtggggtggaaagta	screening
	SLC7A8_ex8_rev	tgggtatcatttgccagatcct	screening
SLC7A8 (MIM604235) exon 9	SLC7A8_ex9_for	tgggttactaggggaggttc	screening
	SLC7A8_ex9_rev	tggagaatggaaatagtggtcc	screening
SLC7A8 (MIM604235) exon 10	SLC7A8_ex10_for	ctttgcccttgaggatgtgt	screening
	SLC7A8_ex10_rev	tctctgggaaaaggcatctg	screening
SLC7A8 (MIM604235) exon 11	SLC7A8_ex11_for	cagaacctggagcaggagag	screening
	SLC7A8_ex11_rev#2	accaaagtcctaccactgcc	screening
p.Arg8Pro	SLC7A8_pArg8Pro__for	gaaggagccaggcacccaaacaacaccgaaaag	cloning
	SLC7A8_pArg8Pro_rev	cttttcggtgttgtttgggtgcctggctccttc	cloning
p.Gly18Arg	SLC7A8_Gly18Arg_for	gaaacacccaggtaggggcgagtcggacgcc	cloning
	SLC7A8_Gly18Arg_rev	ggcgtccgactcgcccctacctgggtgtttc	cloning
p.Ser29Phe	SLC7A8_Ser29Phe_for	cagccccgaggctggtttcggagggggcggagtag	cloning
	SLC7A8_Ser29Phe_rev	ctactccgccccctccgaaaccagcctcggggctg	cloning
p.Ala94Thr	SLC7A8_Ala94Thr_for	ggagccctctgctatactgaactcggggtcacc	cloning
	SLC7A8_Ala94Thr_rev	ggtgaccccgagttcagtatagcagagggctcc	cloning
p.Phe142Ser	SLC7A8_Phe142Ser_for	gtcatcgccctcacctcctccaactacgtgctg	cloning
	SLC7A8_Phe142Ser_rev	cagcacgtagttggaggaggtgagggcgatgac	cloning
p.Met291Ile	SLC7A8_Met291Ile_for	gcttatgtcactgcaatctccccccaggagctg	cloning
	SLC7A8_Met291Ile_rev	cagctcctggggggagattgcagtgacataagc	cloning
p.Val302Ile	SLC7A8_Val302Ile_for	gctggcatccaacgccatcgctgtgacttttggag	cloning
	SLC7A8_Val302Ile_rev	ctccaaaagtcacagcgatggcgttggatgccagc	cloning
p.Ser487Asn	SLC7A8_Ser487Asn_for	ctgctaaccctggtgaaccagaagatgtgtgtg	cloning
	SLC7A8_Ser487Asn_rev	cacacacatcttctggttcaccagggttagcag	cloning
p. Phe436Serfs*22	SLC7A8_Phe436Serfs*22_for	gaccagcaggaagcccagaacagcaag	cloning
	SLC7A8_Phe436Serfs*22_rev	cttgctgttctgggcttcctgctggtc	cloning
Slc16a12 (NM172838)	sense	gctggggctggatgatagt	qRT-PCR
Slc16a12 (NM172838)	anti-sense	tgagcaaagtatgtctgaaactcc	qRT-PCR
Slc16a12 (NM172838)	probe	Universal probe library Roche #38 (Basel, Switzerland)	qRT-PCR
Slc16a10 (NM028247.4)	sense	cgcctacggggtgctcttc	qRT-PCR
Slc16a10 (NM028247.4)	anti-sense	actcacgatggggcagcag	qRT-PCR
Slc16a10 (NM028247.4)	probe	cgagcccacccacgctgtcttg	qRT-PCR
Slc7a8 (NM 016972)	sense	tccacgtttggtggagtcaa	qRT-PCR
Slc7a8 (NM 016972)	anti-sense	tcacacaaccggtactaggt	qRT-PCR
Slc7a8 (NM 016972)	probe	ctccctcttcacctcctcccggct	qRT-PCR
Slc3a2 (NM008577.1)	sense	gtttttgaatgccactggca	qRT-PCR
Slc3a2 (NM008577.1)	anti-sense	gtcctgaggagcgtctgaaa	qRT-PCR
Slc3a2 (NM008577.1)	probe	atggtgcagctggagtgtgtcgc	qRT-PCR
Slc7a11 (AB022345.1)	sense	tggaactgctcgtaatacgcc	qRT-PCR
Slc7a11 (AB022345.1)	anti-sense	ggttccaggatgtagcgtcc	qRT-PCR
Slc7a11 (AB022345.1)	probe	tggagctactgctgtgatatccctggcat	qRT-PCR
Slc7a5 (AB017189.1)	sense	tttgcttggcttcatccagat	qRT-PCR
Slc7a5 (AB017189.1)	anti-sense	ttggacgtbtacttcaacagg	qRT-PCR
Slc7a5 (AB017189.1)	probe	aaggacatgggacaagctgatgcgtc	qRT-PCR
Slc43a2 (NM173388)	sense	gctgattgcatatggagcaagtaac	qRT-PCR
Slc43a2 (NM173388)	anti-sense	cgaagtgaacgtcatgcacat	qRT-PCR
Slc43a2 (NM173388)	probe	ctctctgtgctcatctttatcgccttggc	qRT-PCR
18S rRNA	AppliedBiosystems, #4310893E		

Table S3
Amino acid concentration in serum, aqueous humor and lens from wildtype, TAT1 knock out, LAT2 knock out and TAT1/LAT2 double knockout mice,

	Serum, µM, mean±SD				AH, µM, mean±SD				Lens, nmol/lens, mean±SD			
	WT<br>n=23	TAT1<br>n=7	LAT2<br>n=14	dKO<br>n=8	WT<br>n=23	TAT1<br>n=7	LAT2<br>n=13	dKO<br>n=8	WT<br>n=23	TAT1<br>n=7	LAT2<br>n=14	dKO<br>n=8
Gly	272.01<br>77.63	325.85<br>72.44	283.92<br>130.25	150.48<br>63.46<br>E*	57.05<br>26.42	53.19<br>10.79	70.79<br>37.61	46.43<br>15.25	1.64<br>0.75	2.19<br>0.56	1.66<br>0.83	1.17<br>0.72
Ala	387.03<br>111.18	336.33<br>120.99	411.38<br>188.73	256.30<br>150.79	153.52<br>40.76	109.71<br>24.56	147.44<br>40.99	88.49<br>29.95	2.43<br>0.76	1.89<br>0.41	7.13<br>4.16<br>A***<br>C**	5.76<br>4.17<br>B**D*
Val	175.70<br>54.68	244.40<br>62.32	235.18<br>116.40<br>A**	124.80<br>58.34	92.86<br>33.92	83.23<br>13.51	110.08<br>49.67<br>A*C*	55.75<br>20.84	1.43<br>0.38	1.17<br>0.24	0.78<br>0.45<br>A***C*	0.59<br>0.29<br>B***<br>D**
Leu	114.35<br>33.04	148.93<br>40.33	131.86<br>66.96	80.38<br>53.62	63.43<br>20.81	49.71<br>10.23	61.50<br>25.70	31.85<br>13.56	1.16<br>0.38	0.64<br>0.17	0.67<br>0.36<br>A***	0.46<br>0.26<br>B***
Ile	69.33<br>22.93	100.28<br>22.02	87.08<br>45.42<br>A*	45.78<br>20.23	33.17<br>14.10	29.64<br>5.13	37.18<br>18.37	17.26<br>7.88	0.50<br>0.22	0.28<br>0.09	0.32<br>0.21<br>A***	0.17<br>0.15<br>B***<br>D**
Met	54.60<br>18.65	64.38<br>23.23	63.65<br>30.15	39.59<br>23.96	40.86<br>15.94	26.44<br>2.72	38.59<br>18.08	21.78<br>7.51	0.84<br>0.38	0.39<br>0.10	0.59<br>0.29<br>A***	0.45<br>0.31<br>B***D*
Ser	117.02<br>35.01	130.97<br>49.54	131.53<br>56.35	72.85<br>38.34	83.81<br>23.04	65.19<br>10.84	63.80<br>20.99	37.13<br>15.16	1.30<br>0.51	1.05<br>0.37	2.94<br>1.60<br>A***<br>C**	2.26<br>1.64<br>B*
Thr	134.16<br>32.23	134.18<br>24.48	182.09<br>64.92<br>A**C**	100.55<br>55.03<br>E**	70.22<br>15.21	43.99<br>5.44<br>F***	57.58<br>17.10<br>C*	30.75<br>11.89<br>B***E*	1.05<br>0.38	0.49<br>0.30	1.44<br>0.99	1.05<br>0.87
Pro	91.54<br>34.08	95.13<br>34.85	102.69<br>56.39	68.54<br>57.50	22.35<br>8.32	15.73<br>8.08	26.76<br>10.77	18.70<br>11.29	0.63<br>0.27	0.60<br>0.16<br>F*	0.67<br>0.34<br>C**	0.80<br>0.52<br>D*
Asn	57.62<br>22.31	57.38<br>42.12	67.73<br>21.10	44.44<br>28.31	28.34<br>13.10	19.03<br>10.15	18.23<br>6.13<br>A*	10.28<br>5.83<br>B***	0.19<br>0.14	0.13<br>0.09	0.18<br>0.15	0.17<br>0.14
Gln	683.03<br>161.02	706.26<br>152.62	699.85<br>240.38	346.21<br>85.99<br>E*	426.82<br>111.31	293.01<br>74.35<br>F*	432.96<br>94.00<br>C*	229.53<br>36.40<br>B*D*	5.45<br>2.55	3.64<br>1.37	15.34<br>8.97<br>A***<br>C***	8.74<br>6.27<br>E*
Phe	62.54<br>19.17	133.87<br>49.19<br>F**	51.99<br>18.67<br>C**	68.45<br>49.27	43.42<br>17.72	48.05<br>9.01	20.58<br>9.01<br>A**<br>C***	19.53<br>8.10	1.14<br>0.55	1.11<br>0.47	0.38<br>0.27<br>A***<br>C***	0.47<br>0.22<br>B***<br>D***
Tyr	67.34<br>25.67	351.14<br>110.84<br>F***	73.18<br>24.80<br>C***	221.61<br>110.93<br>B***<br>E***	52.12<br>26.89	128.96<br>33.88<br>F***	27.83<br>9.61<br>C***	69.44<br>18.28<br>B***D*	1.79<br>0.95	3.59<br>0.74<br>F***	0.53<br>0.30<br>A***<br>C***	1.78<br>1.06<br>D***E*
Trp	73.95<br>23.47	214.14<br>45.65<br>F***	71.91<br>17.14<br>C**	80.46<br>29.58<br>B***E*	11.80<br>4.65	21.26<br>4.10<br>F***	5.36<br>2.48<br>C***	6.22<br>2.49<br>D*E*	0.50<br>0.26	0.67<br>0.40<br>F***	0.25<br>0.21<br>A*C***	0.42<br>0.09<br>D***
Lys	240.01<br>69.47	249.42<br>60.78	288.06<br>99.15<br>A*C*	145.29<br>49.32<br>E*	123.81<br>30.15	90.70<br>13.87	144.86<br>29.61<br>A**C***	95.93<br>24.92<br>D*	2.16<br>0.63	1.89<br>0.43	2.10<br>1.46	1.33<br>1.18
Arg	98.78<br>36.25	95.17<br>22.57	94.15<br>34.21<br>C*	49.98<br>17.30<br>E*	28.73<br>12.43	10.88<br>3.78	18.69<br>7.70	11.89<br>3.73	1.60<br>0.51	1.04<br>0.36<br>F*	0.91<br>0.54<br>A***	0.69<br>0.43<br>B***
His	59.69<br>14.37	75.29<br>19.67	50.52<br>16.06	32.81<br>9.84<br>B*D*	34.58<br>10.59	29.45<br>8.45	26.71<br>10.30	15.29<br>3.48<br>B*	0.64<br>0.28	0.39<br>0.11	0.39<br>0.22<br>A***	0.38<br>0.23<br>B***D*
Asp	14.74<br>5.66	14.49<br>8.63	14.42<br>4.28	11.82<br>4.21	18.97<br>8.70	16.58<br>5.88	21.34<br>12.36	14.53<br>3.93	0.67<br>0.31	0.54<br>0.22	0.91<br>0.47	0.68<br>0.54
Glu	51.75<br>34.89	46.26<br>33.91	37.04<br>17.86	33.04<br>11.12	39.56<br>16.39	31.75<br>7.39	58.42<br>30.31	41.72<br>19.40	6.85<br>2.21	6.85<br>1.02	8.04<br>4.63	5.98<br>4.98
Tau	707.91<br>380.00	819.56<br>204.62	527.62<br>217.31	413.68<br>125.27	491.36<br>231.36	467.13<br>112.26	541.52<br>231.93	438.44<br>178.62	80.62<br>30.64	88.91<br>21.60	65.03<br>33.74	40.35<br>33.71<br>B**D**

Table S4
Amino acid concentration in serum, aqueous humor and lens from wildtype, homozygous LAT2 knock out and heterozygous LAT2 knockout mice.

	**Serum, µM, mean±SD**			**AH, µM, mean±SD**			**Lens, nmol/lens, mean±SD**		
	WT n=7	LAT2-KO n=5	LAT2-hetero n=9	WT n=7	LAT2-KO n=4	LAT2-hetero n=8	WT n=7	LAT2-KO n=5	LAT2-hetero n=9
Gly	235.50	355.50	259.88	48.95	65.64	44.05	1.20	1.15	1.29
	36.13	111.86	58.15	15.40	17.03	6.78	0.20	0.61	0.25
		A*				C*			
Ala	469.26	598.45	559.80	149.81	141.15	135.32	2.64	7.69	3.04
	108.99	161.35	290.00	19.84	21.21	25.18	0.56	5.20	0.33
								A**	C*
Val	156.28	246.03	196.42	74.98	87.35	96.23	1.35	0.53	1.68
	26.33	77.98	38.13	13.67	12.70	9.98	0.26	0.35	0.25
		A*				B*		A***	C***
Leu	109.85	155.13	133.00	57.49	56.65	66.81	1.24	0.48	1.36
	13.19	55.03	42.38	4.21	6.81	9.01	0.13	0.30	0.19
								A***	C***
Ile	57.69	92.26	75.86	24.55	28.27	34.66	0.45	0.21	0.67
	8.17	37.56	17.14	6.82	11.18	5.72	0.21	0.15	0.09
		A*						A*	B*C***
Met	62.22	76.66	67.60	44.04	39.27	43.46	1.10	0.56	1.23
	9.93	14.92	42.69	10.66	16.52	7.61	0.20	0.36	0.17
								A**	C***
Ser	124.85	186.55	148.12	81.85	58.49	73.86	1.49	2.68	1.54
	24.11	41.45	62.43	11.90	8.78	13.45	0.38	1.78	0.19
					A*				
Thr	151.95	237.15	176.04	72.46	52.59	70.11	1.25	1.34	1.32
	17.14	40.71	63.97	7.75	15.23	11.22	0.18	1.00	0.25
		A*			A*				
Pro	115.63	150.22	126.74	24.93	27.15	22.15	0.71	0.58	0.77
	15.01	58.06	73.72	1.46	7.49	8.97	0.17	0.37	0.12
Asn	59.81	83.99	64.84	24.41	14.71	19.95	0.22	0.19	0.20
	18.68	18.57	21.17	10.70	1.52	4.77	0.14	0.18	0.06
Gln	785.95	956.79	778.06	488.52	496.57	443.52	6.56	16.91	7.10
	55.78	137.52	106.57	111.58	54.09	57.55	2.36	11.16	1.68
		A*	C*					A*	C*
Phe	59.51	58.22	59.94	44.66	17.33	43.85	1.22	0.19	1.06
	10.85	8.80	11.40	16.29	3.84	7.37	0.40	0.10	0.32
					A*	C*		A***	C***
Tyr	72.73	85.56	70.47	60.71	27.84	56.98	1.99	0.37	1.90
	34.55	15.51	21.06	40.36	5.04	17.76	1.02	0.21	0.58
								A**	C**
Trp	72.23	71.46	72.45	13.26	3.70	12.35	0.60	0.09	0.52
	17.72	18.50	14.50	5.56	0.55	4.56	0.16	0.03	0.13
					A*	C*		A***	C***
Lys	262.04	357.46	272.00	119.42	129.54	110.99	2.28	1.72	2.40
	59.93	67.58	101.49	21.18	28.69	26.24	0.62	1.28	0.37
Arg	96.33	122.07	103.51	29.28	18.09	29.37	1.67	0.70	1.49
	32.51	17.26	36.39	14.31	9.51	5.13	0.35	0.44	0.20
								A***	C***
His	59.97	60.34	57.88	33.04	24.31	34.01	0.71	0.26	0.75
	3.99	7.77	12.21	7.75	4.01	5.04	0.18	0.17	0.10
								A***	C***
Asp	14.83	15.68	14.55	22.85	26.41	22.44	0.68	0.81	0.74
	6.62	3.54	6.01	8.79	13.24	9.42	0.22	0.55	0.17
Glu	58.84	49.32	49.50	42.67	55.75	34.95	7.36	7.85	8.59
	37.96	22.40	13.39	19.32	20.89	9.06	2.01	5.76	1.04
Tau	764.38	666.42	698.17	603.19	553.88	421.23	86.54	51.77	96.50
	384.34	281.19	184.03	309.10	180.53	94.03	18.46	33.06	12.65
								A*	C**
Orn	72.86	71.92	66.74	31.97	28.28	22.74	0.09	0.08	0.11
	28.08	17.07	19.58	12.87	13.58	7.55	0.04	0.06	0.04

Table S5
Statistical assessment of cataract development in the mouse population.

Group	Genotype	No Cataract	Yes Cataract	OR	CI lower	CI upper	P
all	WT	94	3				
	Tat1KO	120	8	2.0889	0.5393	8.0909	0.2863
	Lat2KO	40	8	6.2667	1.5804	24.848	0.009
	dKO	39	35	28.12	8.1635	96.86	<0.0001
young	WT	39	3				
	Tat1KO	36	1	0.3611	0.0359	3.6311	0.3871
	Lat2KO	17	1	0.7647	0.0741	7.8895	0.8217
	dKO	10	7	9.1	1.9892	41.629	0.0044
old	WT	55	0				
	Tat1KO	84	7	9.8521	0.5515	175.99	0.1198
	Lat2KO	23	7	35.426	1.9432	654.84	0.016
	dKO	29	28	107.24	6.3188	1819.9	0.0012
females	WT	42	1				
	Tat1KO	46	5	4.5652	0.5122	40.687	0.1736
	Lat2KO	14	5	15	1.6119	139.59	0.0373
	dKO	21	25	50	6.3326	394.78	0.0002
males	WT	52	2				
	Tat1KO	74	3	1.0541	0.1701	6.5318	0.9549
	Lat2KO	26	3	3	0.4717	19.082	0.2445
	dKO	18	10	14.444	2.8871	72.266	0.0012
young females	WT	11	1				
	Tat1KO	10	1	1.1	0.0605	20.014	0.9487
	Lat2KO	7	1	1.5714	0.084	29.411	0.7623
	dKO	10	7	7.7	0.8006	74.055	0.0772
old females	WT	31	0				
	Tat1KO	36	4	7.7671	0.4023	149.95	0.1747
	Lat2KO	7	4	37.8	1.8293	781.09	0.0187
	dKO	11	18	101.35	5.6376	1821.9	0.0017
young males	WT	28	2				
	Tat1KO	26	0	0.2151	0.0099	4.6901	0.3285
	Lat2KO	10	0	0.5429	0.024	12.266	0.7009
	dKO	0	0	11.4	0.1838	707.19	0.2479
old males	WT	24	0				
	Tat1KO	48	3	3.5361	0.1756	71.223	0.4097
	Lat2KO	16	3	10.394	0.5031	214.75	0.1297
	dKO	18	10	27.811	1.5293	505.75	0.0246

Table S6

SLC7A8 variants in patients with early onset or age-related cataract

Patient ID	exon	age of onset	RNA	Protein	rs	ExAC frequency (European inc. Finnish)	ExAC frequency (total)	SNP database
1	1	late (ARC)	c.23G>C	p.Arg8Pro	141772308	0.001289	0.0008156	0.0008 (GO-ESP)
2, 3, 4, 5, 6	1	late (ARC)	c.86C>T	p.Ser29Phe	149980964	0.004517	0.008044	0.0082 (1000 Genomes); 0.0028 (GO-ESP)
7, 8	2	late (ARC)	c.280G>A	p:Ala94Thr	139927895	0.00189	0.00202	0.0020 (1000 Genomes); 0.0012 (GO-ESP)
9	8	late (ARC)	c.1092A>G	p.Pro364Pro	767062087	no data	no data	no data
10, 11	1	early (congenital)	c.52G>A	p.Gly18Arg	144958980	0.000015	0.001748	
12	1	early (congenital)	c.86C>T	p.Ser29Phe	149980964	0.004517	0.008044	0.0082 (1000 Genomes); 0.0028 (GO-ESP)
13, 14, 15	2	early (congenital)	c.280G>A	p:Ala94Thr	139927895	0.00189	0.00202	0.0020 (1000 Genomes); 0.0012 (GO-ESP)
16	3	early (congenital)	c.487C>T	p.Leu163Phe	novel	no data	no data	no data
17	6	early (congenital)	c.873G>A	p.Met291Ile	141119454	0.0007942	0.0004531	0.0005 (GO-ESP)
18	6	early (congenital)	c.904G>A	p.Val302Ile	142951280	0.0007792	0.0004613	0.0005 (GO-ESP
19, 20	11	early (congenital)	c.1460G>A	p.Ser487Asn	150487356	0.001756	0.001427	0.0018 (1000 Genomes)
21	10	early (congenital)	c.1305del	p.Phe436Serfs*22	778197019 **	no data	no data	no data
